# The Production of Fat-Containing Cultured Meat by Stacking Aligned Muscle Layers and Adipose Layers Formed From Gelatin-Soymilk Scaffold

**DOI:** 10.3389/fbioe.2022.875069

**Published:** 2022-04-12

**Authors:** Chi-Han Li, I-Hsuan Yang, Cherng-Jyh Ke, Chih-Ying Chi, Jefunnie Matahum, Che-Yung Kuan, Nehar Celikkin, Wojciech Swieszkowski, Feng-Huei Lin

**Affiliations:** ^1^ Ph.D. Program in Tissue Engineering and Regenerative Medicine, National Chung Hsing University, Taichung, Taiwan; ^2^ Institute of Biomedical Engineering and Nanomedicine, National Health Research Institutes, Hsinchu, Taiwan; ^3^ Institute of Biomedical Engineering, College of Medicine and College of Engineering, National Taiwan University, Taipei, Taiwan; ^4^ Biomaterials Translational Research Center, China Medical University Hospital, Taichung, Taiwan; ^5^ Faculty of Material Science and Engineering, Warsaw University of Technology, Warsaw, Poland

**Keywords:** cultured meat, *in-vitro* meat, gelatin, soymilk, aligned muscle structure, adipose tissue

## Abstract

Tissue engineered cultured meat has been proposed as an emerging innovative process for meat production to overcome the severe consequences of livestock farming, climate change, and an increasing global population. However, currently, cultured meat lacks organized tissue structure, possesses insufficient fat content, and incurs high production costs, which are the major ongoing challenges. In this study, a developed scaffold was synthesized using gelatin and soymilk to create a friendly environment for myogenesis and adipogenesis in C2C12 and 3T3-L1 cells, respectively. The fat containing cultured meat was fabricated with an aligned muscle-like layer and adipose-like layer by stacking these layers alternately. The muscle-like layer expressing myosin and the adipose-like layer abundant in fat were sandwiched to form fat containing muscle tissue. The cytotoxicity and cell survival rate were evaluated using the WST-1 assay and live/dead staining. Myogenesis was confirmed by the expression of myogenin and myosin. The myotubes, myofibrils, and sarcomeres were observed under an inverted microscope, fluorescence microscope, and scanning electron microscope. Adipogenesis was evaluated by protein expression of the peroxisome proliferator-activated receptor γ, and oil droplet accumulation was determined by fluorescence microscopy with Nile Red stain. Extracellular matrix secretion was examined by safranin-O staining. In this study, the cultured meat was prepared with muscle-like texture with the addition of pre-adipocyte, where the multilayered muscle-like tissues with fat content would produce juicy cultured meat.

## Graphical Abstract

Scheme for cultured meat preparation **(A)** Gelatin–soymilk solution is prepared and then put on the PDMS mold to prepare a GS scaffold with parallel microchannels. **(B)** The C2C12 myoblasts and 3T3-L1 pre-adipocytes are seeded on the GS scaffold to prepare adipocyte-topped muscle-like layers and adipose-like layers. **(C)** The two layers are then stacked layer by layer to assemble into cultured meat containing fat.
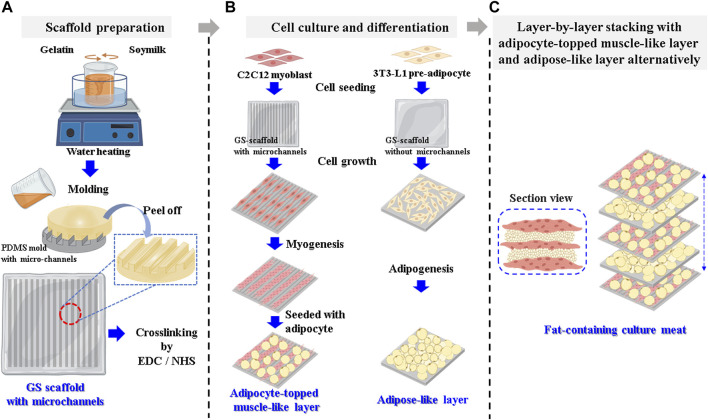

## Introduction

Currently, animal husbandry is the major means of supporting meat demand. However, there are several consequences that trouble the pastoralists, such as production of greenhouse gases, environmental pollution, incidences of zoonotic diseases, excessive animal slaughter, and increased meat demand that is insufficient to match the rising human population (Bhat et al., 2019). Furthermore, huge amounts of natural resources are required for livestock, including global land, freshwater, and antibiotics. Animal husbandry contributes to 15–24% of greenhouse gas emissions, which is higher than the emissions contributed by the global transportation sector (Tuomisto and Teixeira de Mattos, 2011). Some zoonoses can be transmitted from animals to humans through contaminated meat or the environment. The human variant of the Creutzfeldt-Jakob disease (vCJD), which causes brain injury and death transmission through prion contaminated meat products containing, is a well-known example (Bruce et al., 2001; Sanchez-Juan et al., 2007). Animal welfare is a major concern for livestock. In 2014, approximately 65 billion animals were slaughtered. Animal protectionists and religious people are against slaughtering of animals. In 2050, the global population is predicted to rise to nine billion, and the meat demand is predicted to increase to 465 million tons. Population growth is faster than meat produced by domestic animals, and meat demand will likely be a critical issue in the future (Steinfeld et al., 2006; Herrero et al., 2009).

Artificial meat is an alternative to satisfy the demand for meat, which can effectively reduce the use of energy, land, water, as well as reduce greenhouse gas emissions, disease cross transmission, and the number of slaughtered animals. Moreover, reducing the number of close interactions between humans and animals will sharply decrease the development and incidence of epidemic zoonoses. The aseptic techniques employed throughout the culturing process ensures that the meat is free from contamination. Controlled culture conditions can provide meat products with different nutritional, textural, and taste profiles (Tuomisto and Teixeira de Mattos, 2011). Cultured meat production systems have the potential to control meat composition and quality by modifying flavor, fatty acid composition, fat content, and the ratio of saturated to unsaturated fatty acids (Bhat and Fayaz, 2010). In addition, several health boosting and functional ingredients can be added to the meat during formulation to manipulate the flavor (Jones, 2010). Raising animals such as chickens and cows might take months or years; in contrast, cultured meat would ensure rapid meat production to match the growing global population and combat the predicted food shortage (Cassiday, 2018).

Along with the technological development of cultured meat, researchers have been involved in the process of selecting and placing progenitor cells on a well-designed scaffold, growing them in bioreactors, and using cultured cells for muscle tissue production (Arshad et al., 2017). Since Post et al. produced a hamburger from cultured meat, various types of cultured meat have been demonstrably synthesized. However, cultured meat with a structure similar to that of real meat and possessing different tissue composition, comprising mostly adipose cells and aligned muscle cells, is still challenging (Choudhury et al., 2020).

A well-designed scaffold that supports the formation of tissue with muscle-like structure and muscle-adipose tissue composition, while being safe and cost-efficient to manufacture is one solution to overcome the challenges of cultured meat. Various studies have been performed to obtain scaffolds with aligned structures that allow myoblasts to form mature myotubes, including electrospinning (Wang et al., 2015), 3D printing (Kim et al., 2018), and 3D bioprinting (Costantini et al., 2017; Garcia-Lizarribar et al., 2018). In these studies, electrospun nanofibers or anisotropic scaffolds can mimic the natural structure of muscle for myoblast alignment, fusion, and aligned myotube formation. However, these methods are not suitable for the mass production of cultured meat because they are time consuming and require specific equipment.

On the other hand, fat production is an important and often neglected component of most academic studies on cultured meat. Although fat typically accounts for a small part of the total meat content, it is a key component of the texture, nutrition, flavor, and appearance of meat (Fish et al., 2020). Developing edible biomaterial-based scaffolds that provide a highly biocompatible environment for supporting adipose and muscle cell agriculture is an inevitable necessity for the development of cultured meat that incorporates fat.

In this study, we propose a scaffold made up of gelatin and soymilk to synthesize muscle constructs incorporating fat tissue. An ideal scaffold for cultured meat production should provide a biocompatible environment to support the growth and differentiation of muscle and adipose cells, as well as comprise edible biomaterials. Gelatin is a denatured collagen with high biocompatibility and is a common ingredient or additive in the food and pharmaceutical industries (Djagny et al., 2001; Liu et al., 2015; MacQueen et al., 2019). Gelatin contains an abundance of integrin binding sites, thereby improving cell adhesion and migration and eliciting differentiation signaling cues (Nichol et al., 2010; Xiang et al., 2018; Shi et al., 2019). Soymilk, a natural and abundant protein resource, is widely consumed as a soy-based product, such as nutritious protein beverages, soy yogurt, and tofu (Peng et al., 2016). Some meat analogs produced from soybean proteins have been commercially released. Furthermore, scaffolds derived from soy based biomaterials have also proved to possess promising biocompatible properties for cell culture and implantation for tissue regeneration applications (Chien and Shah, 2012; Chien et al., 2013). Soymilk contains bioactive isoflavones, such as daidzein, genistein, and glycitein, which can induce myogenesis. The mixture of genistein, daidzein, and glycitein increased the myotube diameter and the number of myotubes significantly by binding to the endoplasmic reticulum and modulating insulin-like growth factor 1 and major histocompatibility complex expression (Zheng et al., 2018). Daidzein promotes myogenic differentiation and myotube hypertrophy via the promyogenic kinases, Akt and p38, which in turn activate the main myogenic transcription factor MyoD (Lee et al., 2017). In addition, bioactive ingredients present in soymilk, such as 6-hydroxydaidzein (6-HD) and soluble soybean protein peptic hydrolysate (SPH), have been investigated to promote adipogenesis. 6-HD enhances 3T3-L1 adipocyte differentiation by increasing PPARγ gene expression and transcriptional activity (Chen et al., 2013). SPH stimulates lipid accumulation during adipocyte differentiation of 3T3-L1 cells by upregulating PPARγ expression levels (Goto et al., 2013).

Accordingly, the developed cultured meat had a muscle-like texture with pre-adipocyte addition, where the multilayered muscle-like tissues with fat produced tasteful and juicy cultured meat. First, a gelatin–soymilk gel was developed and prepared into a scaffold with parallel microchannels, which would guide myotubes along the long axis of the channels from the fusion of myoblasts and lead to the formation of a single layer of muscle-like structure. Second, the pre-adipocytes were cultured onto the scaffold to develop into an adipose-like layer. Third, the pre-adipocytes were seeded onto a muscle-like layer (prepared in the first step) and then developed into an adipocyte-topped muscle-like layer. Fourth, the adipose-like layer (produced in the second step) and adipocyte-topped muscle-like layer (produced in the third step) would stack layer by layer to form a structure resembling muscle with fat. With the use of tissue-like layers stacking strategy, the incorporating ratio of adipose tissue into the muscle structure can be adjusted allowing the nutrients to be naturally supplied from the organized muscle structure. In this study, we demonstrated that the fat-containing cultured meat with organized muscle structure and sufficient fat content provides an innovative way to overcome the ongoing challenges of cultured meat. The developed scaffold composed of gelatin and soymilk would provide a friendly environment for cell stay, which can turn the cultured cells toward the correct differentiation pathway and drive the cost down. The overall process for the production of cultured meat can be schemed as a graphical abstract.

## Materials and Methods

### Materials and Apparatus

Soybean powder (contain ∼52% protein and 1% fat), gelatin (from porcine skin, Type A, gel strength 300), N-hydroxysuccinimide (NHS), N-(3-dimethylaminopropyl)-N-ethylcarbodiimide (EDC), insulin, dexamethasone, methyl isobutyl xanthine, WST-1 reagent, Safranin-O, Nile Red, and chemiluminescence HRP subtract were obtained from Sigma-Aldrich (St. Louis, MO, United States). Calcein AM, ethidium homodimer-1 (EthD-1), Hoechst 33,342, Alexa Fluor 488 Phalloidin, Dulbecco’s modified Eagle’s medium (DMEM), fetal bovine serum (FBS), and horse serum (HS) were obtained from Thermo Fisher Scientific (San Jose, CA, United States). C2C12 mouse myoblasts and 3T3-L1 mouse pre-adipocytes were purchased from Bioresources Collection and Research Center (BCRC, Hsinchu, Taiwan). Primary antibodies against myosin (MF20), myogenin, glyceraldehyde 3-phosphate dehydrogenase (GAPDH), peroxisome proliferator-activated receptor γ (PPARγ), and secondary antibodies, including goat anti-mouse IgG and horseradish peroxidase (HRP)-labeled goat anti-rabbit IgG were purchased from Thermo Fisher Scientific (San Jose, CA, United States). The fluorescent microscope and confocal microscope were obtained from Leica (Wetzlar, Germany), the fluorescence CCD imager was from GE Healthcare Life Sciences (MA, United States), and scanning electron microscope (SEM) was from Hitachi High-Tech Corporation (Tokyo, Japan).

### Preparation of the Gelatin–Soymilk Solution

Food grade soybean powder (10 g) was suspended in 100 ml double distilled water (ddH_2_O). The suspension was cooked at 100 °C for 1 h with gentle stirring. The cooked suspension was cooled to room temperature (RT), and the insoluble residuals were removed to obtain soymilk. Soymilk (3 ml) was mixed with 20 g of gelatin powder and then diluted to 100 ml using ddH_2_O to prepare gelatin–soymilk solution at 40 °C.

### Preparation of the GS Scaffold

The polydimethylsiloxane (PDMS) mold was imprinted with silicon wafers with parallel microchannels that were patterned using photolithography techniques. The gelatin–soymilk solution (200 μl) was poured onto the PDMS mold at 50°C and then cooled for gelation at –20°C for 8 min. After removing the mold, a GS scaffold (1.5 × 1.0 cm^2^, 80 μm height) with microchannels (100 μm width and 40 μm height) was obtained. An image of the GS scaffold is provided in Supplementary Figure S1. The scaffold was crosslinked with 0.05 M NHS and 0.2 M EDC at RT for 1 h and rinsed with phosphate buffered saline (PBS) to remove excess crosslinking agent. The GS scaffold without microchannels (1 cm^2^) was also prepared using the same molding procedure with a flat PDMS mold.

### Cytotoxicity of the Developed GS Scaffold

The cell viability of the GS scaffold on C2C12 myoblasts and 3T3-L1 was evaluated using the WST-1 assay, based on the guidelines set by the ISO-10993, and briefly described as follows:

The GS scaffold was soaked in DMEM for 24 h, and the extract was cultured with the target cells (C2C12 myoblasts or 3T3-L1 pre-adipocytes) as the experimental group. The cells cultured in DMEM served as the control group, with zinc diethyldithiocarbamate and Al_2_O_3_ extracts used as the positive and negative controls, respectively. After culturing for 3 days, the cells were washed with PBS, and then added to 100 μl of fresh medium and 10 μl of Premix WST-1 that was allowed to react for 30 min at 37°C. The medium was collected from the culture dish and analyzed using an enzyme linked immunosorbent assay (ELISA) reader (Molecular Devices, CA, United States). The absorbance at 450 nm was recorded as the OD value, in terms of cell viability.

### Cell Viability on the Developed GS Scaffold

The cell survival rate of C2C12 and 3T3-L1 cells on the GS scaffold was evaluated using a live/dead staining assay. The cells were cultured on the GS-scaffold. On day 1 and 3, the cells were stained with dyes in the dark at 37°C for 1 h. Living and dead cells were stained with calcein acetoxymethyl ester (calcein AM) and EthD-1, respectively. The cells were observed using a fluorescent microscope, where the living cells were green and dead cells were in red under the excitation wavelengths of 490 and 528 nm, respectively.

### Preparation of the Muscle-like Layer

C2C12 myoblasts were cultured in DMEM supplemented with 10% FBS at 5% CO_2_ and 37°C. C2C12 cells were seeded at a concentration of 2 x 10^4^ onto the GS scaffold in a 6-well plate for 2 days (∼80% confluence). C2C12 cells were then driven toward myogenic differentiation in DMEM supplemented with 2% HS and 100 nM dexamethasone for 14 days. The differentiation medium was changed every 3 days. Myoblasts developed into extensive myotubes and later fused into contracting myofibrils on the scaffold to develop into a muscle-like layer.

### Preparation of the Adipose-like Layer

3T3-L1 pre-adipocytes were cultured and proliferated in DMEM supplemented with 10% FBS at 5% CO2 and 37 °C. 3T3-L1 cells at a concentration of 5 x 10^4^ cells were seeded onto the GS scaffold in a six well plate and driven towards adipogenic differentiation by two kinds of adipogenic media induced in two different stages: adipogenic medium 1 was provided for the first 3 days of culture followed by adipogenic medium 2 for the next 7 days of culture. The adipogenic medium 1 was composed of DMEM supplemented with 10% FBS, 10 μg/ml insulin, 1 μM dexamethasone, and 0.5 mM methyl isobutyl xanthine. Adipogenic medium 2 was composed of DMEM supplemented with 10% FBS and 10 μg/ml insulin. An adipose-like layer was obtained for stacking with a muscle-like layer.

### Preparation of Cultured Meat

3T3-L1 cells at a concentration of 5 x 10^4^ cells were cultured on the muscle-like layer (details in *Preparation of the muscle-like layer*) and then differentiated into adipocytes to be layered on top of the muscle-like layer to form the adipocyte topped muscle-like layer. The differentiation procedure was similar to that described in *Preparation of the adipose-like layer*, but the adipogenic medium 1 was modified to DMEM supplemented with 2% HS, 10 μg/ml insulin, 1 μM dexamethasone, and 0.5 mM methyl isobutyl xanthine, and adipogenic medium 2 was modified to DMEM supplemented with 2% HS, 1 μM dexamethasone, and 10 μg/ml insulin. The adipocyte topped muscle-like layers were alternately stacked with an adipose-like layer as described in *Preparation of the adipose-like layer* and formed into muscle with a muscle-like structure containing fat tissue. The extracellular matrix (ECM) proteins secreted from mature adipocytes act as natural bioadhesives for the stacking of tissue layers.

### Immunostaining of Myosin and F-Actin

The immunostaining of myosin and actin is briefly described as follows. For myosin staining, the muscle-like layer was fixed using 10% formaldehyde in PBS (20 min, RT), rinsed with PBS, and then permeabilized in 0.1% Triton X-100 for 20 min at RT. The membranes were blocked in 1% bovine serum albumin (BSA) (1 h, RT) and incubated in diluted myosin antibody MF20 (1:800 in 1% BSA, overnight, 4°C), followed by rinsing with PBS and incubated with diluted goat anti-mouse IgG secondary antibody (1:5,000 in 1% BSA, 1 h, RT). The layer was then incubated in diluted Hoechst 33,342 solution (1:1,000 in PBS, 30 min, RT) and rinsed with PBS. The layer was first examined under a fluorescence microscope and further examined using a confocal microscope. For F-actin staining, the muscle-like layer was fixed, permeabilized, and blocked as described previously with myosin staining. The layer was then incubated in diluted Alexa Fluor 488 phalloidin (1:40 in PBS, 30 min, 37 °C). Finally, the layer was incubated in diluted Hoechst 33,342 solution (1:1,000 in PBS, 3 min, RT). Gross observations were performed using a fluorescence microscope, and an in depth examination was performed under a confocal microscope.

### Analysis of the Myotubes Along the Microchannel

Myosin stained fluorescent images were analyzed using ImageJ software to measure the myotube length and angle shift from the long axis of the microchannel. Data were collected and measured from 20 myotubes in each image.

### Western Blot Analysis

C2C12 cells were trypsinized from the muscle-like layer and lysed using a lysis buffer, followed by centrifugation at 12,000 rpm for 15 min to obtain protein lysates. The protein lysates were loaded onto a 10% SDS-polyacrylamide separation gel, electrophoresed, and transferred to a polyvinylidene difluoride (PVDF) membrane. The membrane was blotted first with 1% BSA to decrease non-specific binding of the primary antibody. The membrane was immunoblotted with a primary antibody, followed by incubation with an HRP-conjugated secondary antibody. Immunoreactive blots were revealed using a chemiluminescence-HRP subtract, and images were captured using a digital fluorescence CCD imager (Wang et al., 2016).

Adipocytes were trypsinized from the adipose-like layer, lysed, and centrifuged (12,000 rpm, 15 min) to obtain protein lysates. The following processes of electrophoresis, protein transfer to PVDF, and immunoblotting were similar to the previous process, except for the primary antibody PPARγ (Lin et al., 2021).

### Safranin-O Staining

Safranin-O, a cationic dye, was designed for the detection of acidic proteoglycans present in the ECM. Muscle-like layers were fixed with 4% paraformaldehyde at RT for 20 min, soaked in 0.1% acetic acid for 15 s, and stained with 0.1% Safranin-O solution for 30 min. The layers were rinsed thrice with PBS. The acidic proteoglycans in the ECM were observed using an inverted microscope.

### Nile Red Staining

Nile Red staining was carried out to detect intracellular lipid droplets and was examined by fluorescence microscopy. The adipose-like layers were fixed with 4% paraformaldehyde at RT for 20 min, and then incubated in 0.5 μg/ml Nile Red solution at 37 °C for 30 min. The layers were rinsed with PBS three times and then observed using a fluorescence microscope under excitation between 550 and 640 nm.

### Scanning Electron Microscopy

SEM was used to visualize the repeating sections of the sarcomeres in the myofibrils. The well-differentiated myofibrils on the scaffold were mounted on the sample stage and coated with a gold film by ion sputter. Silver glue was spotted onto the peripheral area to discharge free electrons to enhance image quality. The stage was mounted on the SEM to observe myofibril morphology.

### Statistics

All quantitative data are expressed as mean ± standard error of the mean. Statistical analysis was performed using the Student’s *t-test* to analyze variance. For the determination of statistical significance, *p-values* smaller than *0.05* were considered to be significant.

## Results

### Cell Viability and Survival Rate on the Developed GS Scaffold

**FIGURE 1:**
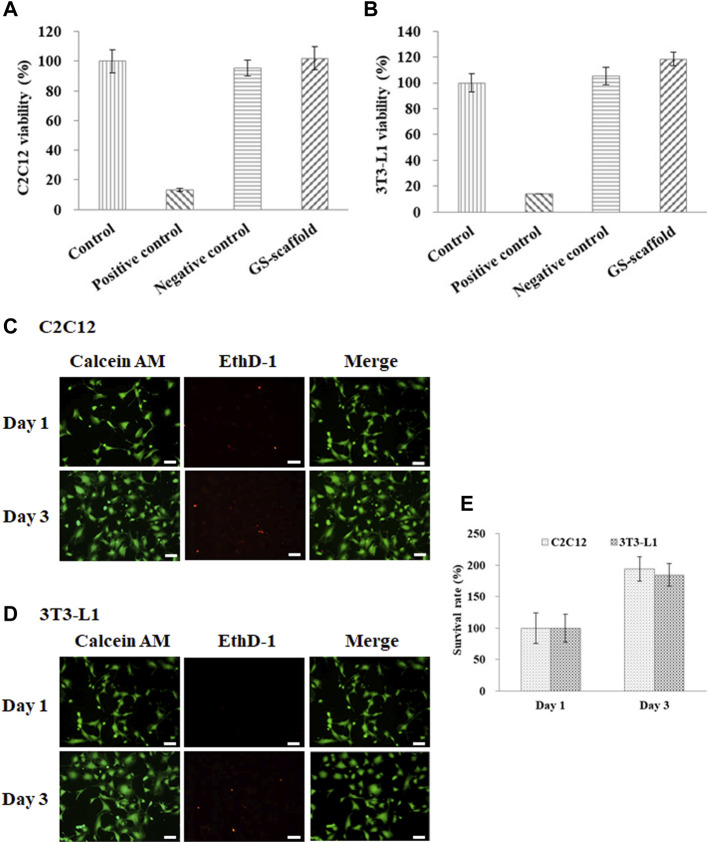
Cell viability and survival rate of the **(A)** C2C12 and **(B)** 3T3-L1 cells were evaluated based on the guidelines provided by the ISO-10993. The results are in terms of cytotoxicity. The cells cultured with medium served as the control group, where the cells cultured with the extracts from ZDEC and Al_2_O_3_ were used as the positive and negative controls, respectively. Data are presented as the mean ± SD, n = 6, in two independent experiments. The cell viability of the **(C)** C2C12 and **(D)** 3T3-L1 cells on the GS scaffold were evaluated by live/dead staining. Cells were grown on the GS scaffold and analyzed at day 1 and 3. The living cells in green were stained with Calcein AM, and the dead cells in red were stained with EthD-1 (scale bar = 50 μm). **(E)** The quantification of the C2C12 and 3T3-L1 cells by live/dead staining.

In this study, gelatin and soymilk were used as raw materials to prepare scaffolds to sustain C2C12 and 3T3-L1 cultures. Cell viability was determined using the WST-1 assay, based on the guidelines put forth by ISO-10993. As shown in Figure 1, the optical density (OD) value of the control group was set to 100% cell viability. The cell viability of C2C12 cells in the negative control (Al_2_O_3_) and the positive control (ZDEC) were 95.4 ± 5.1 and 13.3% ± 0.9%, respectively. The cell viability of C2C12 cells on the GS scaffold was 102.1 ± 7.6%, as shown in Figure 1A. Figure 1B shows the results of cell viability of 3T3-L1 cells on the GS scaffold, which was approximately 118.2 ± 5.2%. The cell viability of 3T3-L1 cells in the negative control and the positive control was 105.2 ± 6.8% and 14.1 ± 0.2%, respectively. There was no significant difference in cell viability between the control and the experimental groups. We found that the GS scaffold did not hinder the proliferation of the C2C12 and 3T3-L1 cells. The survival rate of the C2C12 and 3T3-L1 cells cultured on the GS scaffolds was examined by live/dead staining shown in Figures 1C,D, where more than 99% of the C2C12 and 3T3-L1 cells were alive on the scaffolds at day 1 and 3. Furthermore, the cell count on day 3 was much greater than that on day 1 shown in Figure 1E.

The results demonstrated that a scaffold constructed with gelatin and soymilk is suitable for studying myoblast and pre-adipocyte cell adhesion and proliferation. The general features of gelatin as a safe, edible material, and comprising an abundance of integrin binding sites for improving cell adhesion indicated that these scaffolds can support a variety of adherent cell types with a utility for cultured meat production.

### Cell Differentiation on the Developed GS Scaffold

**FIGURE 2:**
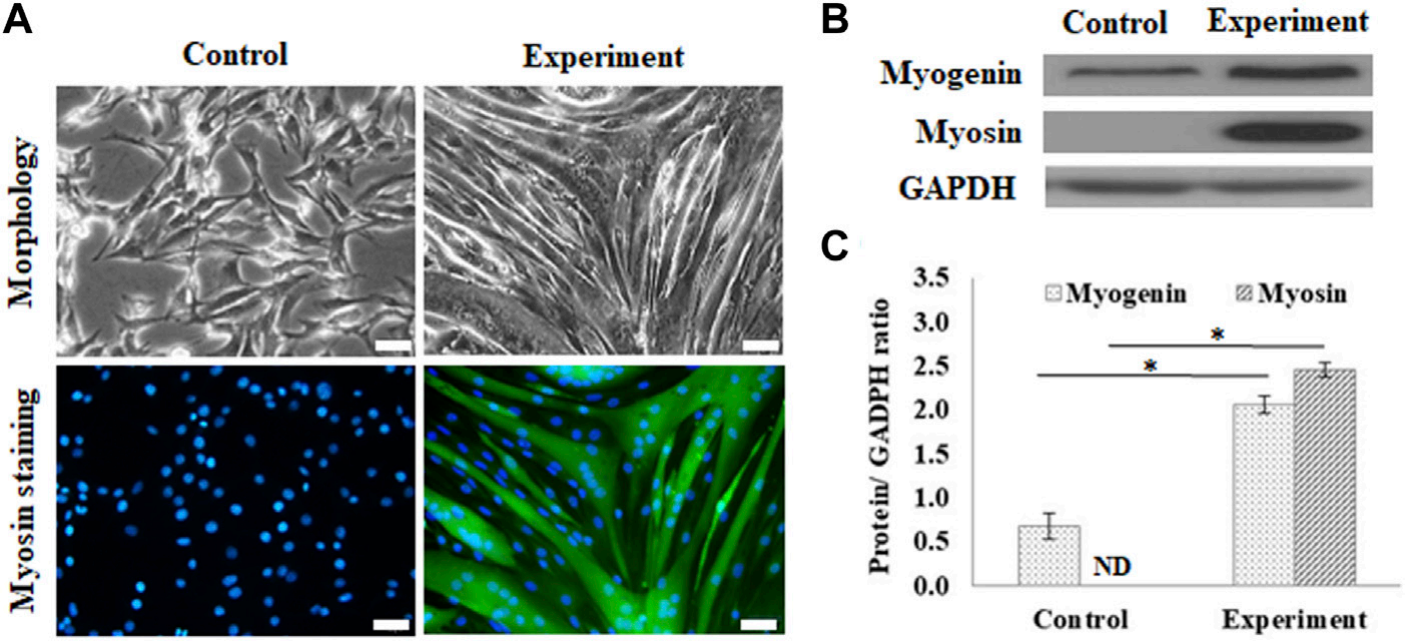
Myogenic differentiation on the GS scaffold without channels. **(A)** The morphology and myosin immunostaining of the differentiated C2C12 cells after 7 days of induction (scale bar = 50 μm). **(B)** The western blots of myogenin, myosin, and GAPDH. **(C)** The quantification of myogenin and myosin expression via the ratio to GAPDH using ImageJ software. The control group comprised cells cultured in the growth medium. Data are presented as the mean ± SD, n = 3, three independent experiments, *p < 0.05, ND is not detected.

The myogenic differentiation observed in C2C12 myoblasts on GS scaffolds was evaluated by myotube formation and the expression of myogenin and myosin, as shown in Figure 2. The control group was cultured in a growth medium, and the experimental group was cultured in a myogenic medium. The myotube formation in C2C12 cells was demonstrated after 7 days of differentiation by myosin staining in green and the nucleus contrast stain in blue, as shown in Figure 2A. On day 7, C2C12 cells changed their morphology from a fibroblast-like spindle shape to a myotube-like appearance and expressed abundant myosin, a protein required for myotube formation.

Figure 2B shows the results of the protein expression of myogenin and myosin, where GAPDH, housekeeping protein, was treated as the reference. Western blotting showed that differentiated cells upregulated myogenic signaling factors, including the transcription factor, myogenin, and the downstream protein, myosin, compared to the control. The calculated data showed that myogenin expression was significantly enhanced in differentiated cells (2.06 ± 0.09 times) compared with the cells in the control group (0.66 ± 0.14 times) (*p* < 0.05). The myosin expression was also significantly enhanced from a nondetectable level in the control group to 2.45 ± 0.08 (*p* < 0.05), as shown in Figure 2C.

**FIGURE 3:**
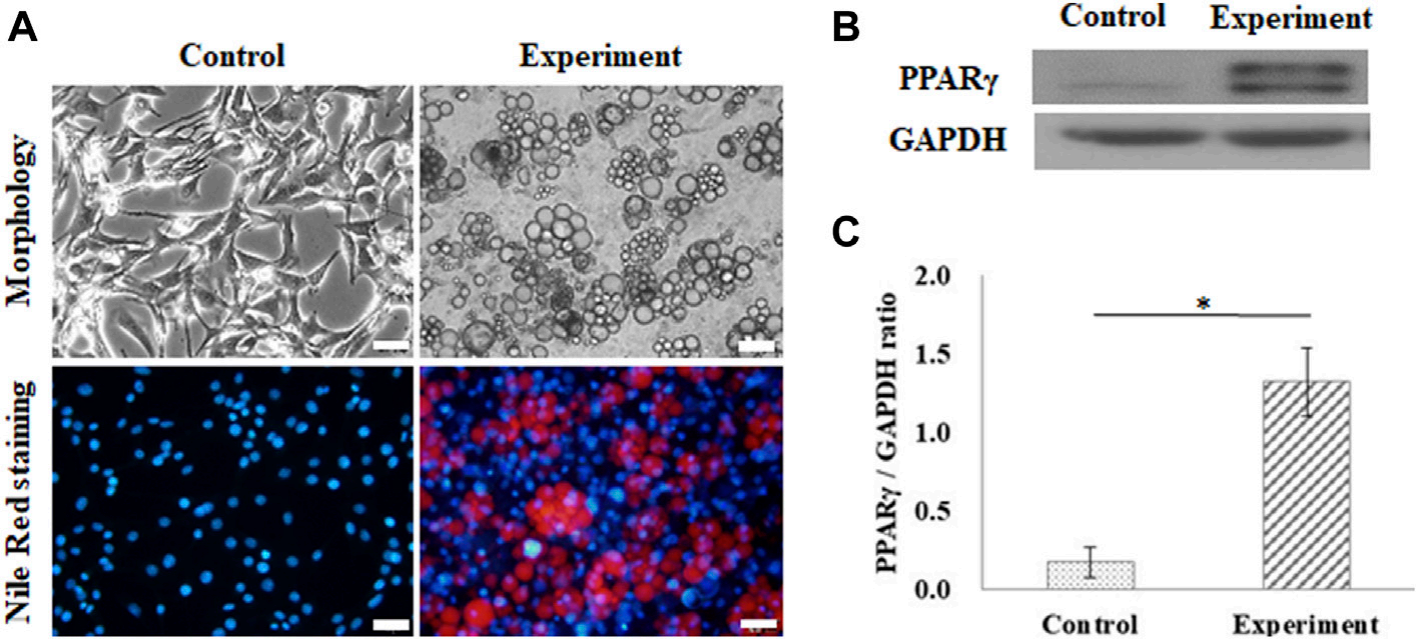
Adipogenic differentiation on the flat GS-scaffold. **(A)** The morphology and Nile Red staining of the differentiated 3T3-L1 cells after 10 days induction (scale bar = 50 μm). **(B)** The western blots of PPAR-γ and GAPDH. **(C)** The quantification of the PPAR-γ expression via the ratio to GAPDH using ImageJ software. The control group comprised cells cultured in the growth medium. Data are presented as the mean ± SD, n = 3, in three independent experiments, *p < 0.05.

Adipogenesis of 3T3-L1 pre-adipocytes on the GS scaffold was also evaluated by lipid accumulation and expression of PPARγ, as shown in Figure 3. The control group was cultured in a growth medium, and the experimental group was cultured in an adipogenic medium. After 10 days of differentiation, the cells induced lipid droplet accumulation in the cytoplasm, which was stained with Nile Red, as shown in Figure 3A. Figure 3B shows the results of the expression of PPARγ in differentiated adipocytes on the GS scaffold, where GAPDH was the reference protein. Western blotting results showed that differentiated adipocytes upregulated the expression of the adipogenic transcription factor PPARγ compared to the expression in the control group, as shown in Figure 3B. The calculated data showed that PPARγ protein expression was significantly enhanced in differentiated cells (1.32 ± 0.09 times) compared with the control group (0.17 ± 0.21 times) (*p* < 0.05), as shown in Figure 3C.

The results indicated that the GS scaffold effectively supported myogenesis and adipogenesis in C2C12 and 3T3-L1 cells, respectively.

### Microchannel-Guided Myotube Formation

**FIGURE 4:**
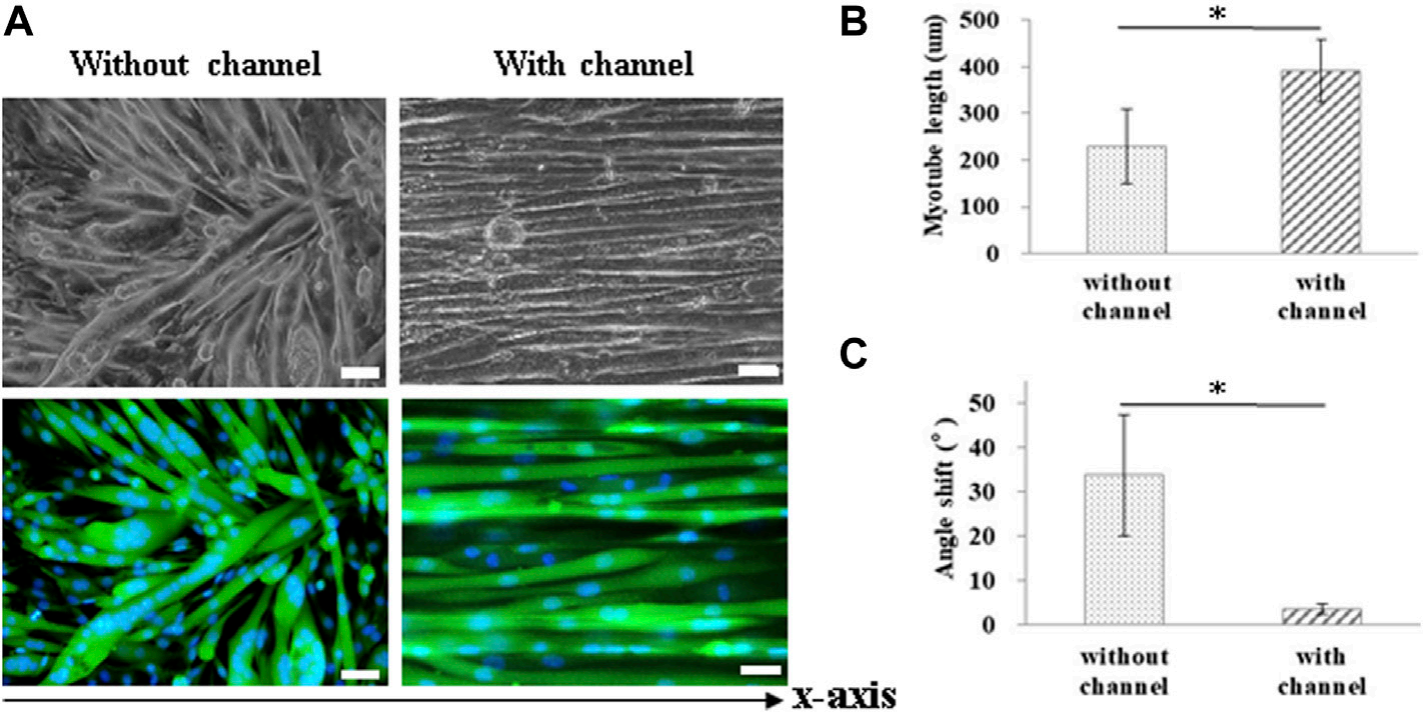
Measurement of myotube alignment on micro channeled GS scaffold. The myotubes were aligned along the micro channels 7 days after differentiation. **(A)** The morphology of the myotubes and myosin immunostaining (scale bar = 50 μm). The quantification of the **(B)** myotube length and **(C)** angle shift using ImageJ software. The control group comprised cells cultured on the GS scaffold without microchannels. Data are presented as the mean ± SD, n = 20, in two independent experiments, *p < 0.05.

For muscle-like layer establishment, a GS scaffold with parallel microchannels was fabricated to promote C2C12 alignment and myoblast differentiation. As shown in Figure 4, the guidance of microchannels for growing myocytes to form unidirectionally aligned myotube monolayers was examined by inverted microscopy. After 7 days of culture in a differentiation medium, C2C12 cells were stained to observe myosin, a specific myogenic protein for myotube formation, and counterstained for observing the nuclei with Hoechst 33,342. Here, the GS scaffold without channels was used to assess the contribution of the microchannel to myotube alignment. The data showed that myotube alignment was significantly higher on channel surfaces than on the surface without the channels, as shown in Figure 4A. As shown in Figures 4B,C, the effects of topographic cues on myotube formation, including the length and angle of myotubes, were quantified based on fluorescent images. Myosin positive myotubes were evaluated by measuring the whole length and the angle shift between the long axis of the myotube and the *x*-axis direction using ImageJ software. Statistical analysis showed that myotubes on the microchannels pattern with significantly longer length (391.0 ± 66.6 μm) compared with those on the unpatterned surface (229.3 ± 80.2 μm) (*p* < 0.05). The statistical analysis also showed that the myotubes on the microchannel pattern had significantly lower angle shift (3.61 ± 1.19°) compared with unpatterned surfaces (33.73 ± 13.64°) (*p* < 0.05). The calculated data indicated that the presence of microchannels on the scaffold provided contact guidance to the growing myocytes, allowing them to form unidirectionally aligned muscle-like layers. In this study, we report that a GS scaffold with parallel microchannels efficiently promotes aligned C2C12 myoblast differentiation. Myoblast differentiation under guidance with microchannels has the capacity to create engineered muscle-like tissues.

### Maturation of the Myotubes

**FIGURE 5:**
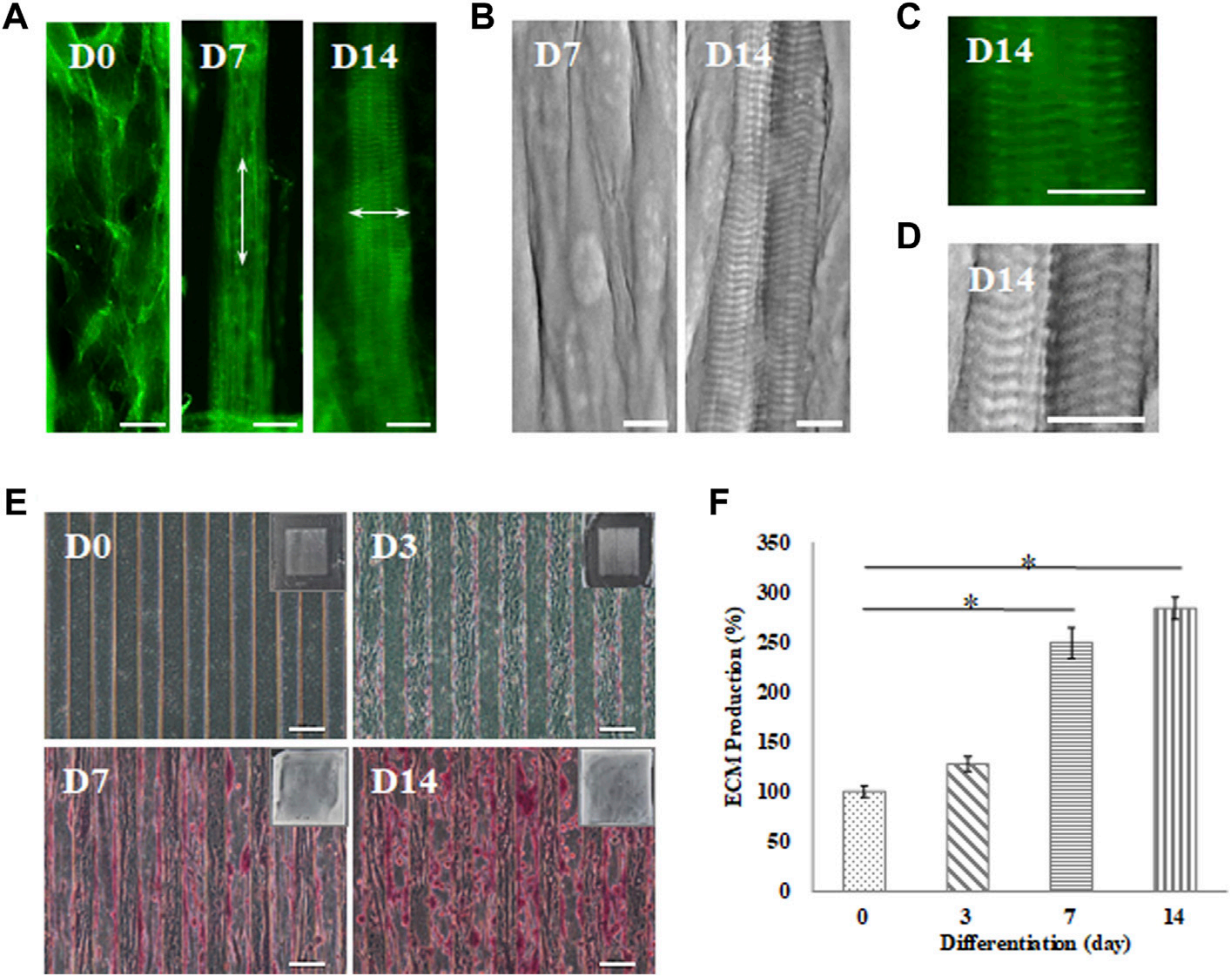
Myotube maturation and ECM secretion demonstrated by the **(A)** myofibrils observed under an inverted microscope with F-actin staining and **(B)** sarcomere examination by SEM on day 7 and 14; the arrow indicates the direction of the actin fiber. Higher magnification was used to examine the **(C)** F-actin stain by inverted microscope and **(D)** sarcomere structures recorded by SEM (scale bar = 30 μm). **(E)** Brightfield imaging and safranin-O staining were used to examine ECM secretion at day 0, 3, 7 and 14 (scale bar = 100 μm); **(F)** The quantification of the positive safranin-O staining area compared to the total area of the image using ImageJ software. Data are presented as the mean ± SD, n = 3, in three independent experiments, *p < 0.05.

The sarcomere structure in the myofibrils was evaluated as an indicator of maturation, as shown in Figure 5. Cells that were induced toward myogenic differentiation on the GS- scaffold at different time stages were stained with phalloidin, a convenient probe for detection of actin filaments observed with fluorescence microscopy. The data showed that myoblasts differentiated into myotubes with actin filaments along the long axis of myotubes after 7 days of induction. Furthermore, myotubes matured into myofibrils and exhibited repeating sarcomere structures in the differentiated myofibrils after 14 days of induction, as shown in Figure 5A. The sarcomere structures of the mature myofibrils were also observed by SEM. The myograph of the myofibrils showed well-organized sarcomeres in the mature myofibrils, as shown in Figure 5B. The magnified images from phalloidin staining observed with SEM show a clear parallel pattern of sarcomeres, as shown in Figures 5C,D. A video further proved the formation of mature myofibrils on the GS-scaffold, which exhibited real time contraction under electrical stimulation (Supplementary Video S1). These data illustrated that well-developed myofibrils on the scaffolds possessed organized sarcomeres and contractive activity.

In this study, C2C12 myoblasts cultured on the GS scaffold for 0, 3, 7, and 14 days were observed under an inverted microscope to examine ECM formation by safranin-O staining, as shown in Figure 5E. The data demonstrated that myofibrils secreted higher levels of ECM in a time-dependent manner. Quantification of the positive safranin-O staining area related to the total area of the image showed that ECM secretion increased by 28% on day 3, increased by 149% on day 7, and increased by 183% on day 14 compared to the value on day zero (*p* < 0.05), as shown in Figure 5F. In our data, mature myofibrils in the muscle-like layer were composed of repeating functional sarcomere structures and secreted abundant ECM.

### Formation of the Engineered Muscle Construct and Adipose Tissue *via* the Stacking of the Tissue Layers

**FIGURE 6:**
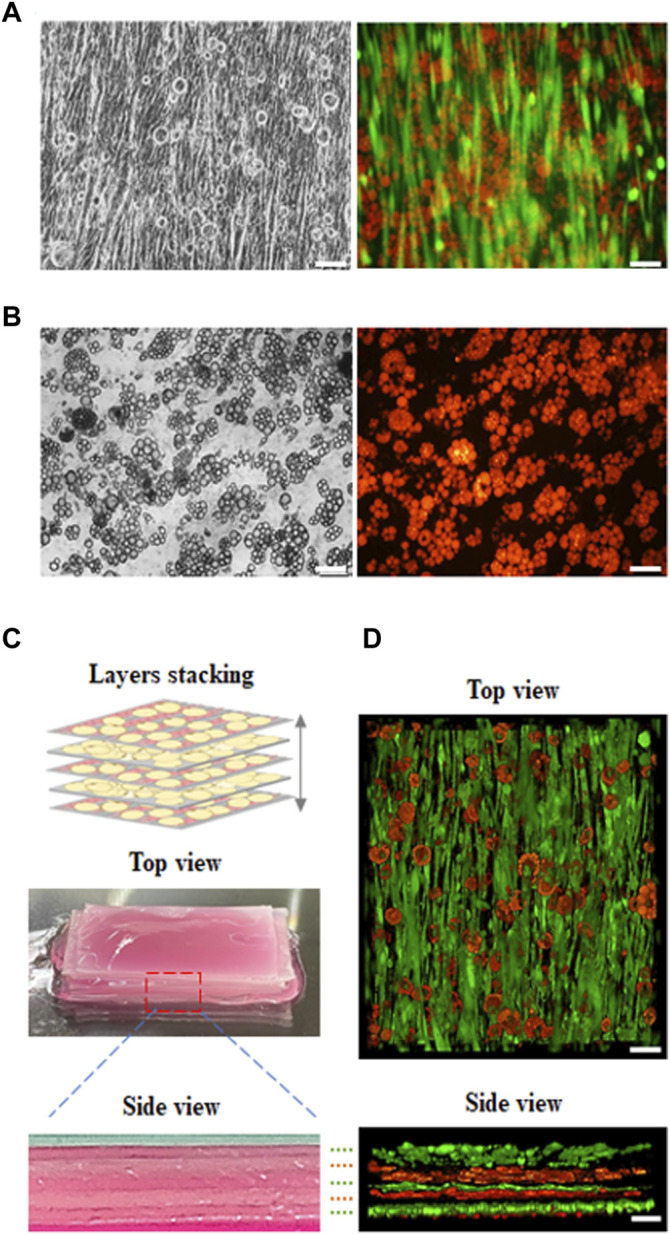
Fluorescence images of developed cultured meat. The cultured meat containing fat was synthesized with a **(A)** muscle-like layer topped with adipocytes, with myosin (green) and lipid droplets (red); **(B)** adipose-like layer with lipid droplet (red). **(C)** The previous two layers (a, b) were made by alternately stacking them to develop cultured meat containing fat as shown in the digital camera images (top and side view). **(D)** The 3D view in z-stack reconstruction (top and side view) of cultured meat containing fat (scale bar = 100 μm).

In our study, cultured meat containing fat was synthesized using an aligned muscle-like layer and an adipose-like layer using layer by layer stacking. First, C2C12 myoblasts were seeded on the microchannels in the GS scaffold and cultured for 14 days to form a muscle-like layer. The layer was then seeded over with 3T3-L1 pre-adipocytes to turn into a muscle–adipocyte layer, as shown in Figure 6A. Second, the 3T3-L1 pre-adipocytes were seeded on the GS scaffold and cultured for 10 days to form the adipose-like layer, as shown in Figure 6B. Third, three muscle-adipocyte-mixed layers and two adipose-like layers were stacked alternately to develop into an engineered muscle construct with an aligned myofibril structure with incorporated adipose tissue, as shown in Figure 6C. The different layers were stacked with sticky ECM secreted from mature adipose tissue to form a solid construct. The cross-sectional image showed that the layers were closely integrated. The fluorescent 3D images in Figure 6D show that the muscle-like layer maintained structural integrity, and the adipose tissue layer was also maintained with abundant fat accumulation. Furthermore, using this stacking strategy, the incorporation ratio of adipose tissue into the muscle structure can be adjusted by varying the number of muscle or adipose-like layer combinations.

Supplement data further proved the ECM secretion of matured adipose tissue on the GS scaffold. The adipose-like layer was stained with collagen type I, Nile Red, and counterstained with Hoechst 33,342. The data showed that mature adipose tissue with abundant lipid droplet accumulation was immersed in the ECM layer immunostained with collagen type I (Supplementary Figure S2).

A video rotation of a 3D reconstructed confocal z-stack of cultured meat containing fat is also provided to prove the distribution of aligned muscle layers and adipose-like layers (as shown in Supplementary Video S2).

## Discussion

An appropriate scaffolding approach to facilitate cell proliferation and differentiation into essential cell types possessing spatial arrangements that resemble the natural meat structure and texture is required. To date, scaffolds used in cultured meat research are predominantly composed of animal-derived collagen and gelatin. Collagen is a limited resource and thus is not a sustainable way to produce cultured meat. Gelatin, an alternative source, is a denatured collagen with high biocompatibility and contains an abundance of integrin binding sites for improving cell adhesion and migration, and eliciting differentiation signaling cues. Plant-derived biomaterials, including polysaccharides and proteins, are inexpensive, widely available, and can be processed into scaffolds that are suitable for cultured meat production. Plant-derived polysaccharides, such as cellulose, alginate, and agarose, possess good biocompatibility but lack cell recognition sites for cell growth. Plant-derived proteins, such as soy or zein, which are abundant protein resources, have shown promising biocompatibility in cell culture and implantation for tissue regeneration applications (Seah et al., 2021). In our study, the developed GS scaffolds provided a biocompatible environment for cell adhesion, proliferation, and differentiation of myoblasts and pre-adipocytes. The biocompatibility of the GS scaffold provides a possibility for the establishment of muscle-like and adipose-like layers to be stacked into muscle constructs. The materials properties were also characterized by swelling test, degradation test, and rheometry. (Supplementary Figures S3 and S4).

The structure of the native muscle tissue, which consists of unidirectionally aligned myotubes, plays a role in generating contractile forces. Thus, the formation of large numbers of aligned myotubes in a unit area is a key issue in cultured meat production. Various previous studies have demonstrated that structural control promotes the alignment and elongation of cells and enhances the differentiation and maturation of myotubes, including employing techniques like photomold patterning (Ahadian et al., 2012; Hosseini et al., 2012; Ramon-Azcon et al., 2012; Ostrovidov et al., 2017; Fernandez-Garibay et al., 2021), electrospinning (Wang et al., 2015), 3D printing (Kim et al., 2018), and 3D bioprinting (Costantini et al., 2017; Garcia-Lizarribar et al., 2018). However, these methods are not suitable for the mass production of cultured meat because they are time-consuming and require specific equipment. In our study, we used a master model with parallel microchannels for imprinting the GS scaffold replicas without specific equipment; moreover, the method provides fast patterned-scaffold synthesis that is easy to scale up, and benefits batch production to realize commercialization in the future. Our data demonstrated that muscle cells can be aligned and mature on gelatin and soymilk, which effectively mimics the native structure and replicates the biological functions of muscle tissue in cultured meat production.

Cultured meat was developed based on *in vitro* cell culture and tissue engineering. The cells proliferated and differentiated on the scaffold to form multinuclear myotubes. Furthermore, the maturation of myotubes resulted in the formation of muscle fibrils, and the further growth of muscle fibrils resulted in a product that mimics meat (Kadim et al., 2015; Gaydhane et al., 2018). The maturation from myotubes to myofibrils with contractile function is the most important indication of the establishment of a functional muscle-like layer. Mature myofibrils with contractile function consist of repeating sections of the contractile units referred to as sarcomeres, which appear as alternating dark and light bands under the microscope. Shortening or contraction of the sarcomeres leads to the contraction of myofibrils and subsequently, of the muscle as a whole (Scime, 2009; Ostrovidov et al., 2014). The ECM is composed of a 3D network of extracellular macromolecules. The ECM in skeletal muscle is not only considered a structure providing mechanical support and force transmission, but also an appropriate environment for muscle development and functioning (Gillies and Lieber, 2011). In our data, well-developed myofibrils in the muscle-like layer had organized sarcomere structures, contractive activity, and abundant ECM secretion.

Finally, a controllable ratio of muscle and adipose cultured meat can be fabricated by alternately stacking multilayers with muscle-like and adipose-like layers. Previous academic studies have focused on innovations for scalable muscle tissue culture; however, fat content in cultured meat is an important factor that affects flavor, taste, nutrition, and visual appearance. Developing suitable biomanufacturing strategies for adipose tissue from agriculturally relevant animal species may be particularly beneficial because of the potential use of cell cultured fat as a novel food ingredient (Stanford and Goodyear, 2018; Fish et al., 2020). Our study provides an insight into the production of cultured meat where the optimal ratio between muscle and lipid can be achieved by alternately stacking muscle-like and adipose-like layers.

## Conclusion

In this study, a GS scaffold was successfully synthesized using gelatin and soymilk to create a favorable environment for myogenesis and adipogenesis in C2C12 and 3T3-L1 cells, respectively. Meat-like tissue containing fat was synthesized with aligned muscle-like and adipose-like layers by alternately stacking them. The aligned muscle-like layer expressing myosin and the adipose-like layer with abundant accumulated fat were sandwiched between each other to form muscle-like tissue containing fat. We believe that the proposed method would have great potential in the market as a low cost and a relatively quick method of producing cultured meat in the near future.

## Data Availability

The original contributions presented in the study are included in the article/Supplementary Material, further inquiries can be directed to the corresponding author.

## Supplementary Material

Supplementary Figure S1 Image of the GS scaffold and cell growth. **(A)** Digital camera image of a GS scaffold (1.5 cm^2^) with parallel microchannel structure (channel diameter: 100 μm). **(B)** Magnifying image of micro-channels under the microscope (top and side views). **(C)** C2C12 myoblasts were cultured and proliferated in microchannel. **(D)** The differentiated myotubes were aligned along the microchannel. Fluorescent images were used to examine cell growth **(E)** and myotube formation **(F)** on the scaffold (top view and side view). The myosin in green was stained with myosin immunostaining. (scale bar = 100 μm)

Supplementary Figure S2 Extracellular matrix of adipose-like layer was demonstrated by **(A)** digital images were used to examine extracellular matrix secretion on day 0 and 10 (top and side view). **(B)** Fluorescent image of extracellular matrix. The collagen in green was stained with collagen immunostaining, and the differentiated adipose colored in red was stained with Nile Red (top and side view).

Supplementary Figure S3 Swelling and degradation test. In swelling test, the prepared scaffolds were immersed in medium at 37°C. At day 0, 1 and 3, the scaffolds were removed from the medium, wiped dry with filter paper to remove excess medium from the surface, and weighed. **(A)** Digital images were used to examine scaffolds swelling on day 0, 1 and 3 (top and side view). **(B)** The weights of scaffolds were presented as the mean ± SD, n = 3. In degradation test, the prepared scaffolds were immersed in medium at 37°C. On day 0, 1 and 3, the scaffolds were removed from medium, washed with deionized water and dried for 1 h at 60°C. **(C)** Digital images were used to show dry scaffolds on day 0, 1 and 3. **(D)** The prepared scaffolds were weighed for dry weight. Data are presented as the mean ± SD, n = 3.

Supplementary Figure S4 Viscosity analysis. **(A)** Rheological properties of the gelatin-soymilk solution were evaluated at 25°C using a rheometer (Discovery hybrid rheometer-1, TA Instrument.) with 40 mm parallel plate. **(B)** Viscosity was calculated at 60 s in the steady state using a controlled flow mode. Viscosity of the samples significantly increased after cross-linking.

Supplementary Video S1 Video of mature myofibrils developed on the GS-scaffold was captured under a microscope in the bright field. The video further proved the formation of mature myofibrils that exhibited real-time contraction under electrical stimulation.

Supplementary Video S2 Video rotation of a 3-D reconstructed confocal z-stack of fat-containing cultured meat stained with anti-myosin antibody (green) and Nile Red staining (red). The ImageJ 3D viewer was used to generate the projection of stacking from all five tissue-like layers, showing the distribution of muscle-like layers and adipose-like layers.
