# Supplementary figures and images for: The Production of Fat-Containing Cultured Meat by Stacking Aligned Muscle Layers and Adipose Layers Formed From Gelatin-Soymilk Scaffold
Source: Front Bioeng Biotechnol. 2022 Apr 12;10:875069. doi: 10.3389/fbioe.2022.875069 (PMC9039213; doi:10.3389/fbioe.2022.875069)

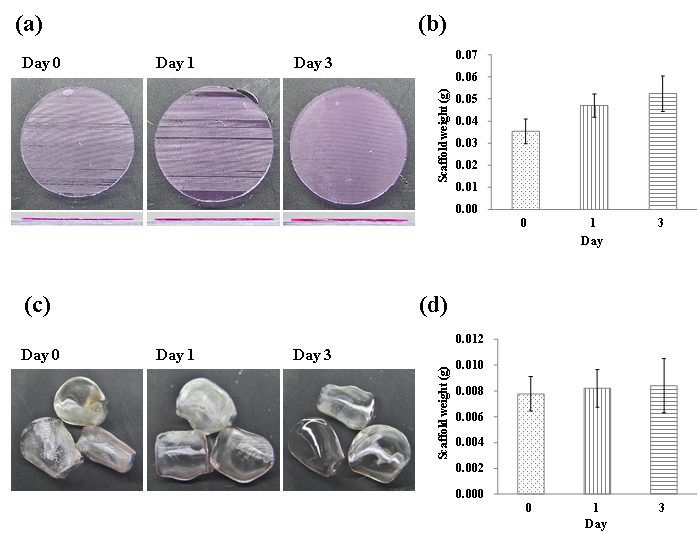

Supplement: Supplementary file 1 [file Image3.TIF]

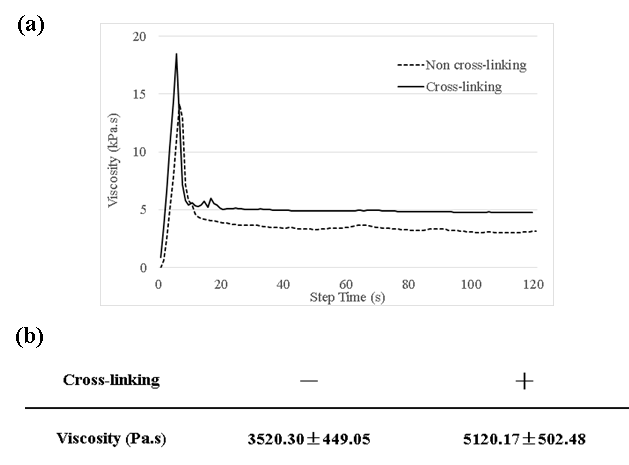

Supplement: Supplementary file 2 [file Image4.TIF]

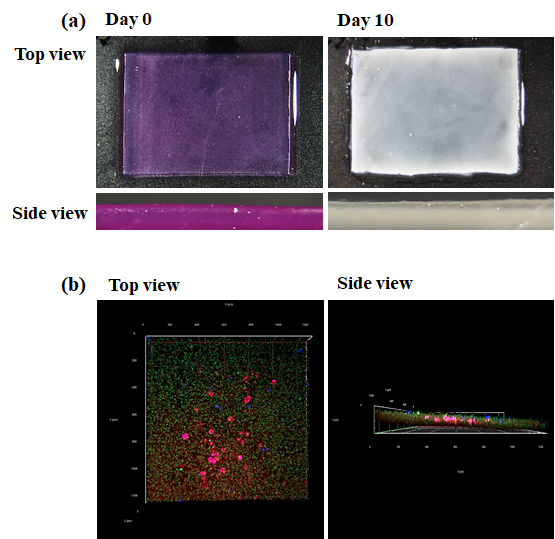

Supplement: Supplementary file 3 [file Image2.TIF]

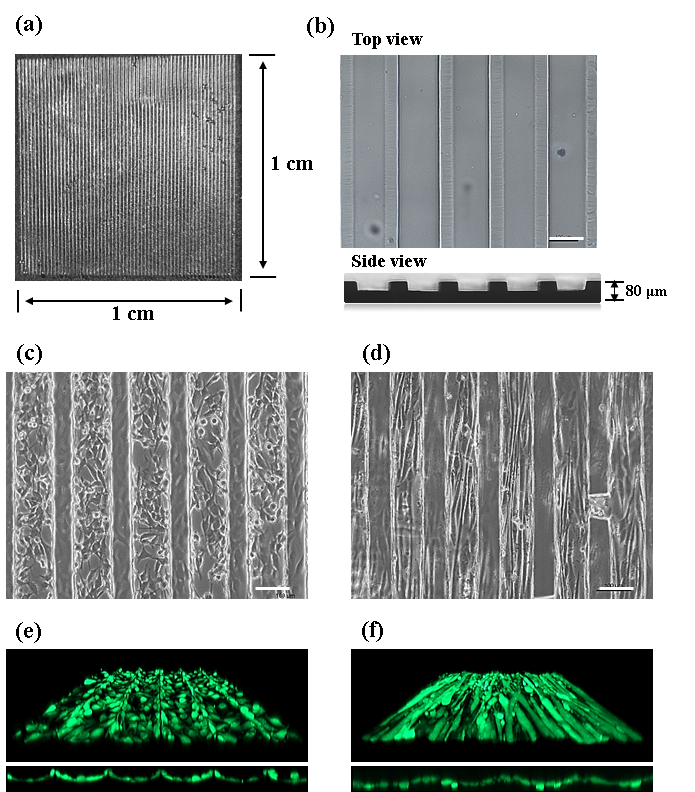

Supplement: Supplementary file 4 [file Image1.TIF]
